# Near-Infrared Spectroscopy as a Tool for Marine Mammal Research and Care

**DOI:** 10.3389/fphys.2021.816701

**Published:** 2022-01-17

**Authors:** Alexander Ruesch, J. Chris McKnight, Andreas Fahlman, Barbara G. Shinn-Cunningham, Jana M. Kainerstorfer

**Affiliations:** ^1^Neuroscience Institute, Carnegie Mellon University, Pittsburgh, PA, United States; ^2^Sea Mammal Research Unit, University of St Andrews, St Andrews, United Kingdom; ^3^Fundación Oceanogràfic de la Comunitat Valenciana, Valencia, Spain; ^4^Kolmården Wildlife Park, Kolmården, Sweden; ^5^Department of Biomedical Engineering, Carnegie Mellon University, Pittsburgh, PA, United States

**Keywords:** near-infrared spectroscopy, marine mammals, physio-logging, wearable, vital signs, diving physiology

## Abstract

Developments in wearable human medical and sports health trackers has offered new solutions to challenges encountered by eco-physiologists attempting to measure physiological attributes in freely moving animals. Near-infrared spectroscopy (NIRS) is one such solution that has potential as a powerful physio-logging tool to assess physiology in freely moving animals. NIRS is a non-invasive optics-based technology, that uses non-ionizing radiation to illuminate biological tissue and measures changes in oxygenated and deoxygenated hemoglobin concentrations inside tissues such as skin, muscle, and the brain. The overall footprint of the device is small enough to be deployed in wearable physio-logging devices. We show that changes in hemoglobin concentration can be recorded from bottlenose dolphins and gray seals with signal quality comparable to that achieved in human recordings. We further discuss functionality, benefits, and limitations of NIRS as a standard tool for animal care and wildlife tracking for the marine mammal research community.

## Introduction

The field of “biologging” (i.e., logging/transmission of biological variables via devices attached to animals) has largely focused on describing where animals go in space and time. The rate of instrumentation development, data collection, and development of analytical approaches in the field of “physio-logging” (i.e., using attached sensors to log physiological variables such as heart rate, blood oxygen content and breathing frequency), however, has evolved at a much slower rate – arguably decades behind disciplines such as ecology (Fahlman et al., 2021a). Yet, continuous physiological measurements provide vital baseline information about the physiology, physiological capacity, and variability in freely moving animals, as well as the physiological responses of organisms to disturbances, including anthropogenic activity. With technological development we now have smaller and lighter telemetry and physio-logging devices with larger storage capacities and longer-lasting batteries, providing the capacity to measure high-resolution physiological parameters over extended periods.

One such technology involves measuring cardiovascular and cardiorespiratory dynamics in animals with continuous-wave near-infrared spectroscopy (CW-NIRS). CW-NIRS is a non-invasive and wearable light-based technology that provides continuous measures of concentration changes in oxygenated hemoglobin (ΔHbO), and deoxygenated hemoglobin (ΔHbR) (Jobsis, 1977; Quaresima and Ferrari, 2019) in tissue. ΔHbO and ΔHbR can provide high temporal resolution measurements of relative blood volume changes, mixed arterial–venous, tissue-specific hemoglobin oxygen saturation (tissue saturation index, TSI), arterial oxygen saturation (SpO_2_), as well as heart rate and cardiac waveforms. We recently demonstrated the utility of CW-NIRS in voluntarily diving seals and breath-hold diving humans performing deep dives (>100 m) (McKnight et al., 2019, 2021a,b). Thus, this technology promises to open new avenues for physiological research in free-ranging animals, as highlighted in a number of recent physio-logging reviews (Fahlman et al., 2021a; Ponganis, 2021; Williams et al., 2021; Williams and Hindle, 2021; Williams and Ponganis, 2021).

Prior to NIRS, electrocardiography (ECG) has been used for decades on various free-ranging species to monitor heart rate (Butler and Woakes, 1980; Thompson and Fedak, 1993; Trondrud et al., 2021). Similarly, blood oxygen measurements in free-ranging animals such as seals, sea lions and penguins, pioneered by Paul Ponganis and colleagues, have been achieved with great success using intravascular-dwelling Clark-type pO_2_ electrodes (Ponganis et al., 2007; Meir et al., 2009; McDonald and Ponganis, 2013). However, a single CW-NIRS device enables extraction of a breadth of physiological data along with a capacity to measure tissue-specific blood volume and oxygenation changes in freely moving animals. Combining NIRS’ non-invasive nature, relatively small size, and the possibility of integrating it into existing animal-borne dataloggers and telemetry systems, makes CW-NIRS a promising technology to develop for physio-logging research.

In this perspective, we outline the functionality and optical theory for how CW-NIRS works, and then demonstrate its feasibility by translating approaches developed for human measurements to different marine mammal species. Further, we provide comparable example physiological data extracted from NIRS signals across species and suggest future research perspectives, considerations, and advice for its application as a research tool in physio-logging research.

## Near-Infrared Spectroscopy

**FIGURE 1:**
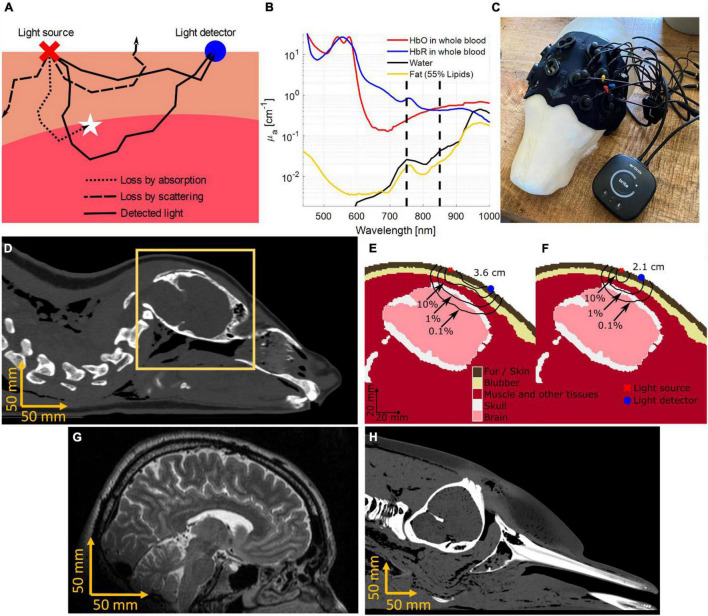
The working principle of Near-Infrared Spectroscopy (NIRS). **(A)** Light interacts with molecules in biological tissue in two main ways, namely scattering and absorption. Scattering affects the direction of light progression, indicated by the black lines, making it possible for light to reach deeper into the tissue and return to the surface. Most scattered light is lost (dashed lines), however, a certain percentage of the light is scattered into the detector (solid lines). Additionally, some light loss can be attributed to absorption (dotted line). **(B)** The absorption spectrum of oxygenated (HbO) and deoxygenated (HbR) hemoglobin are shown under the assumption of 150 g of hemoglobin in 1 L of whole blood. Other common molecules present in biological tissue, such as lipids inside fat and blubber, as well as water emphasize the strong absorption of hemoglobin species for near-infrared wavelengths. Dashed lines indicate wavelengths at 750 and 850 nm, which are often used in medical, recreational, and research CW-NIRS devices. **(C)** The Brite NIRS device (Artinis Medical Systems, Elst, Netherlands) shown on a 3D model of a juvenile gray seal (*Halichoerus grypus*). The neoprene cap was custom fit for optimized optode placement around the brain. **(D)** The cross-section of a harbor seal (*Phoca vitulina*) computer tomogram (CT). **(E)** The orange window in panel **(D)** is segmented into optically relevant tissues. The black lines indicate the probability density of light received at a detector 3.6 cm away from the source (McKnight et al., 2019, 2021b). **(F)** The shorter source detector distance (2.1 cm) has a lower percentage of photons returning from the brain tissue but will in return receive more light. **(G)** A magnetic resonance image (MRI) showing the distance from the scalp to the brain in an adult human. **(H)** A CT scan of a stranded bottlenose dolphin, showing a much longer distance to the brain as compared to humans and harbor seals.

Near-infrared spectroscopy, at its core, relies on two fundamental components – light emission and light detection. NIRS devices emit light of multiple wavelengths into biological tissue by placing LEDs, or optical fibers connected to lasers, onto the skin. An optical detector then quantifies the light that has traveled through tissue at a specified distance from the source. Because biological tissue is approximately 100 times more likely to change the trajectory of a photon (termed scattering) than converting its energy into heat (termed absorption), the light diffuses inside the tissue (see Figure 1A). Therefore, NIRS not only allows transmission measurements (such as a finger probe pulse oximeter), but also measurements in which source and detector reside on the same side of the tissue, achieving sensitivity to tissue layers multiple centimeters below the surface. This is referred to as diffuse reflectance. Using optical properties of biological tissues known from literature (Jacques, 2013), including its light absorption spectrum (Figure 1B), light propagation and sensitivity to tissues can be predicted mathematically (Dehghani et al., 2009). The most likely light propagation path through tissue in a diffuse reflectance setup (such as seen in Figure 1C) resembles a parabola or “banana” shape (see Figures 1D–F).

To measure proportional changes of oxygenated hemoglobin saturation in biological tissue, oxygen rich hemoglobin (HbO) needs to be distinguished from hemoglobin with reduced oxygen concentration (HbR). In simple terms, the oxygenation of blood affects the blood’s color, shifting it from a bright red to a darker blue tinted red as oxygen saturation decreases. Like pulse-oximetry, commonly used in hospitals and sports-oriented smart devices to measure arterial oxygen saturation, NIRS shines multiple wavelengths of light in the red to near-infrared range (typically 700–900 nm) to resolve the color of blood. The color change is caused by differences in the absorption spectra of HbO and HbR (see Figure 1B).

To measure changes in the concentration of HbO and HbR, we first need to measure the change in the absorption coefficient of light (Δμ_a_(λ)) at a minimum of 2 wavelengths (λ). The modified Beer-Lambert’s law (Eq. 1) describes how changes in light intensity (ΔI(λ)), relative to the intensity at time 0 of a measurement (I_0_(λ)), can be translated into Δμ_a_(λ) (Sassaroli and Fantini, 2004; Bigio and Fantini, 2016).


(1) $$\Delta \,\mu_{\text{a}}\,\left(\lambda \right)=\frac{-1}{\text{DPF}\cdot \text{r}}\,\frac{\Delta \,I\,\left(\lambda \right)}{{\text{I}}_{0}\,\left(\lambda \right)}$$

(2) DPF=3μs(λ)′2⁢μa⁢(λ)

Here, r is the distance from the light source to the detector on the surface. The differential pathlength factor (DPF, see Eq. 2) is a function of absolute optical properties of absorption (μ_*a*_) and reduced scattering coefficient ($\mu_{\text{s}}^{\prime }$), known from literature or measurable with more advanced NIRS devices. It relates the average distance traveled of light through the tissue, to r. A typical value for human measurements of the head is DPF ≈ 6. In this example, a 1 cm increase in r will result in a 6 cm increase in average photon travel distance through the tissue. Longer source-detector separation therefore reduces the measured light intensity but yields a deeper average photon penetration depth. This creates a tradeoff between measurement depth and signal strength (compare Figures 1E,F).

Knowledge of the molecular light extinction coefficient (ε) of HbO and HbR from literature (Prahl, 1999; Figure 1B) can be used in a linear equation system (*Eq*. 3) to solve for the concentration changes of all contributors to light absorption (Δ*c*_*n*_), given


(3) $$\Delta \,\mu_{\text{a}}\,\left(\lambda \right)=\sum_{\text{n}}\varepsilon_{\text{n}}\,\left(\lambda \right)\cdot \Delta \,{\text{c}}_{\text{n}}$$

With two wavelengths of light, as used in the examples here, only two absorbers can be distinguished, i.e., n = {HbO, HbR}. In this case, it is common to assume that other absorbers, such as water, lipids, and melanin, are largely constant over the measurement period and can thus be disregarded.

Other NIRS modalities utilizing light pulses or sinusoidally modulated light, as opposed to continuous illumination, can measure absolute optical properties (μ_*a*_, $\mu_{\text{s}}^{\prime }$) leading to absolute HbO and Hb measurements, at a cost of added complexity and reduced mobility. They are described elsewhere (Fantini and Franceschini, 2002; Bigio and Fantini, 2016).

## Practical Approaches to Determine Feasibility of Near-Infrared Spectroscopy in Marine Mammals

**FIGURE 2:**
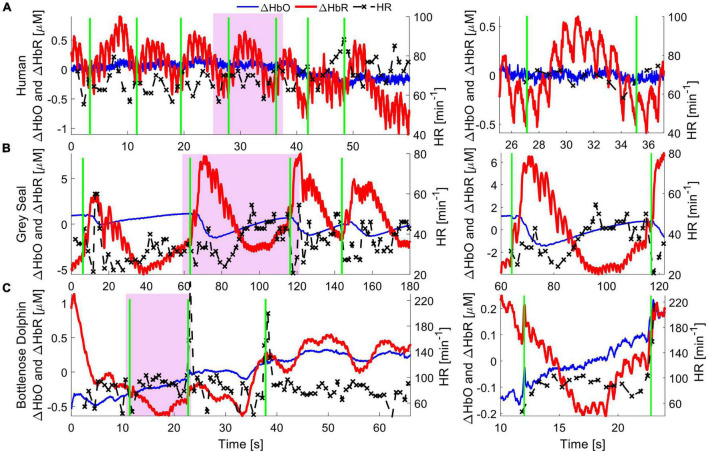
Hemodynamics of cardiac and pulmonary activity. Hemoglobin concentration changes are demonstrated for respiration events in a human (top), gray seal (middle) and bottlenose dolphin (bottom). Oxygenated hemoglobin (red), dominantly present in arteries, shows strong cardiac pulsation. Deoxygenated hemoglobin (blue) is mostly present in capillaries and veins, and only in small concentrations in the pulsating arteries, and thus shows very little pulsation. The green vertical lines indicate manually recorded respiration onsets, be it inhalation in humans or exhalation in marine mammals. The apparent pulse-to-pulse heart rate is shown in black and reveals respiratory sinus arrythmia (RSA) in the human. The seal shows a reduction in heart rate immediately following respiration, alongside an increased hemoglobin concentration pulse amplitude. The bottlenose dolphin shows an increase in heart rate following the respiration event with a slight delay, likely due to RSA. A zoomed in image of the purple shaded areas for single respiration events is shown on the right, to emphasize the cardiac pulsation signal.

As a proof of concept, we compared signals recorded with NIRS between humans and two marine mammals. We measured cardiac pulsation and respiratory events in one human (male, 30 years of age), one bottlenose dolphin (*Tursiops truncatus*, male, 17 years of age), and one juvenile gray seal (*Halichoerus grypus*, male, 2–3 years of age) (Figure 2).

Human experiments were conducted after the participant provided informed voluntary consent. All procedures were approved by the Carnegie Mellon University Institutional Review Board (STUDY2015_00000113). The participant was equipped with a NIRS sensor (Imagent 2.0, ISS Inc., Champaign, IL, United States) above the visual cortex, at the back of the head. After the room was darkened by closing curtains and dimming room lights, the region of interest was illuminated with light at 755 and 808 nm, at a source-detector separation of 3 cm. The participant was asked to remain still and breath at a constant rate for a duration of 2 min. Cardiac pulsation and respiration events recorded from the brain are clearly identifiable in the recorded data, with ΔHbO showing cardiac pulsation (Figure 2A).

For NIRS measurements on the gray seal, wild-caught juvenile animals were temporarily held in a managed facility, before release back to the wild. Procedures for capture, handling and housing of animals conformed to the Animals (Scientific Procedures) Act 1986, under the Sea Mammal Research Units’ Home Office license (no. 70/7806) and were performed by personnel deemed competent under EU directive. During anesthesia, the animals were fitted with a continuous-wave NIRS device (Brite 24, Artinis Medical Systems, Elst, Netherlands). The device was adapted by custom designing a neoprene cap that matched the contour of a juvenile gray seal’s head, holding light sources and detectors to improve skin contact and block interfering room light (McKnight et al., 2021b). The source-detector separation was set to 3 cm. The head was illuminated using LEDs at 750 and 850 nm. To improve the coupling of the light source and receiver to the skin, the animal was shaved locally at each optode position. The original study shows how NIRS can be used to observe functional brain activation resulting from auditory, visual, and tactile stimuli. Here, we specifically observed systemic hemodynamic signals, such as cardiac pulsation and respiration, indicating good NIRS signal quality. The respiratory signal shows that ΔHbO and ΔHbR trend in opposite directions immediately after a respiratory event. This indicates a change in blood flow, leading to a rapid oxygenation of the brain tissue, consistent with rapid and deep respiration events that are common in gray seals (Figure 2B). For more details on the data collection, see (McKnight et al., 2021b).

Finally, we measured a dolphin in a managed-care facility which voluntarily beached itself onto a poolside ledge. A customized CW-NIRS system was then placed on the chest (Custom design of PortaLite, Artinis Medical Systems, Elst, Netherlands). The dolphin remained still and was free to breath of its own volition and return to the water at any time. The experiment was approved by the Oceanogràfic Animal Care & Welfare Committee at the Fundación Oceanogràfic Valencia, Spain (OCE-10-20), and the Bureau of Medicine (BUMED, NRD1170). We illuminated the tissue with light of 760 and 850 nm at an increased source-detector separation of 4 cm to measure light that had reached deeper layers, at the expense of reduced overall light intensity. A rubberized black fabric was placed over the NIRS device and the chest to block interfering sunlight. The hemodynamic changes during cardiac pulsations and respiration observed in the muscle layers indicate a qualitatively comparable signal strength to human and gray seal hemodynamic measurements (Figure 2).

## Discussion

Overall, we achieved similar signal quality and observed similar patterns of change across the cardiac cycle for human and seal measurements of the brain (cortex). Further, the observed signal strength and patterns of change from muscle tissue on the chest in dolphins suggests that CW-NIRS can be applied successfully in these large mammals, despite their thick layer of blubber. Preliminary results, such as those shown in Figure 2, and previous applications (McKnight et al., 2019, 2021a,b) indicate that CW-NIRS can operate across different mammalian species, highlighting its potential as a useful comparative eco-physiological monitoring tool. Because NIRS is wearable and non-invasive, it can be used for animals held in professional care, chemically restrained, and wild animals. However, we suggest that application of NIRS in wild, free-ranging animals is of particular interest. Just as ECG and intravascular-dwelling pO_2_ electrodes have transformed fundamental physiological monitoring capacity through measurement on free-ranging animals, we believe NIRS has similar potential.

### Continuous-Wave Near-Infrared Spectroscopy Perspective

We propose that NIRS may play an important role in understanding gas management and cardiovascular-cardiorespiratory-cerebrovascular regulation in free-ranging animals, which will provide information on energetic regulation. Investigating these fundamental physiological attributes in free-ranging animals is important to understand the capacity and plasticity of diving animals, which may help to better understand the impact of man-made disturbances and environmental change. Our results provide evidence that NIRS can detect both respiration events and heart rate, providing a methodological basis for assessment of cardio-respiratory coupling. For instance, respiratory sinus arrythmia (RSA), seen as variation in heart rate and stroke volume following a breath, has been proposed to improve ventilation-perfusion matching and improve gas exchange (Fahlman et al., 2020b; Blawas et al., 2021). Cardiorespiratory coupling, combined with NIRS derived estimates of arterial blood and tissue oxygenation, allows us to determine cardiac and pulmonary health. This allows us to assess the importance of RSA in gas management of marine mammals, and provides a powerful tool to evaluate how conditioned changes in cardiac function is used to manage blood and tissue gas levels (Fahlman et al., 2019, 2020a,b). In addition, NIRS allows an assessment of how stress may alter gas exchange, which provides vital information to understanding the impact of disturbance on wild animals (Fahlman et al., 2021b). Another potential application we forecast is in cognitive neuroscience. Animals in human care can be trained to participate in cognitive studies in which we can measure the hemodynamic response to neuronal activation using functional-NIRS (fNIRS) (Pinti et al., 2020; McKnight et al., 2021b). Learning how animals process information, react to visual, auditory, and other stimuli, and undertake decision making will aid informed decisions for animal conservation.

There are numerous direct benefits of NIRS over alternative devices, such as electroencephalography (EEG), ECG, and blood draws, including its portability, spatial resolution, and the breadth of systemic and tissue-specific physiological parameters that can be measured. Some NIRS devices can be run off battery power and yet record continuously for hours. The signal created from light shone into the tissue is non-invasive, non-ionizing electromagnetic radiation – considered less harmful than sunlight. The spatial resolution of NIRS is on the order of centimeters and therefore allows assessment of the metabolic requirements of discrete tissues. The hemodynamics of the brain can be compared to hemodynamics on peripheral tissues (such as muscle tissue) or any other perfused body part within a few cm of the skin surface. Bilateral measurements or measurements of disease-affected tissue compared to that in healthy parts of an animal’s body can be used to observe and assess lesions or tumors, especially important in observation and quantification of recovery (Obrig, 2014; Boezeman et al., 2016; Yang et al., 2019; Fantini and Sassaroli, 2020).

### Limitations

As with all devices, CW-NIRS is not free of limitations. The signal quality of NIRS varies with the percentage of light received in the detector after scattering and absorption in the target tissue. Therefore, tissue composition influences signal quality. Measuring the brain in a juvenile gray seal, for example, is possible with commercial instruments built for humans as the brain is close to the scalp (approx. 15 mm based on computer tomographic (CT) scans) (Figures 1D,G). However, in bottlenose dolphins, the light needs to penetrate a thick layer of blubber (approx. 20 mm), bone (up to 15 mm), and, dependent on the measurement location, muscle tissue (up to 20 mm) before making functional measurements of the brain (>50 mm in adult bottlenose dolphins, see Figure 1H); this makes measurements more challenging. If the muscle tissue is the target, then existing NIRS works well in bottlenose dolphins. Measuring the muscle layer of larger cetaceans, such as killer whales, will be more challenging due to the thick blubber layer. However, even in larger species, skin perfusion of blood could be measured to provide cardiovascular information. Increasing the source-detector distances and light intensity may help overcome some of these limitations.

In pinnipeds and other species, fur density can prevent direct skin contact, causing substantial amounts of light to be lost through surface reflection. Of course, this challenge can be overcome by shaving the animals locally (McKnight et al., 2019) or by designing light guides like combs that can reach through the hair. This problem is common with humans and recent devices may allow translation to marine mammals (Khan et al., 2012; Pollonini et al., 2016; Yücel et al., 2020). It must be emphasized that continuous reliable contact is essential for the acquisition of viable NIRS data, and particular care must be taken to promote development of this aspect of CW-NIRS application along with electronic hardware.

### Analysis

The history of NIRS in medical applications has driven development of numerous free tools to analyze data and extract the information relevant to the experiment (Aasted et al., 2015; Santosa et al., 2018; Hou et al., 2021). This includes tools to reduce movement artifacts stemming from insufficient or variable contact between hardware and the skin surface – a phenomenon that is a common challenge in NIRS. Analytical methodologies such as short-channel or accelerometer-based regression (Robertson et al., 2010; Virtanen et al., 2011) and time derivative distribution repair algorithms (Fishburn et al., 2019) in human applications assist in removing motion-artifacts. Short-distance regression can additionally be used to remove the skin perfusion signal from a measurement of deeper tissues such as the brain.

### Hardware Optimization

In addition to open-source tools, modern NIRS device designs (e.g., abandoning glass-fiber guided light in favor of direct placement of light emitting diodes and light sensitive photodiodes on the skin), simplify NIRS devices in a way that make them feasible for new applications like aquatic physio-logging, in which movement is encouraged and devices can be made salt water resistant. Future research and development must further optimize aspects of the NIRS hardware, including, but not limited to, the choice of wavelengths and source-detector separations, attachment mechanisms, battery longevity, and ease of customization for various marine species. For example, low absorption coefficients paired with high scattering coefficients in blubber tissue can make the use of longer wavelengths of light (>1,000 nm) beneficial. Longer wavelengths of light are less energetic and thus less likely to scatter, which can help overcome blubber tissue more easily. However, reduced contrast between molecular extinction coefficients of HbO and HbR could trade deeper penetration for lower signal quality. The optical properties of biological tissue are dependent on the concentration of water, lipids, hemoglobin species, and others, yet assumptions made here are based on terrestrial mammalian tissue (Jacques, 2013). The validation of optical properties of *marine* mammal tissues are needed to make realistic mathematical models that allow optimization of the device properties.

## Data Availability Statement

The datasets presented in this study can be found in online repositories. The names of the repository/repositories and accession number(s) can be found below: Figshare Repository: Data Figure 2 in: Near-Infrared Spectroscopy as a Tool for Marine Mammal Research and Care, https://doi.org/10.6084/m9.figshare.17019668.

## Ethics Statement

The studies involving human participants were reviewed and approved by the Carnegie Mellon University Institutional Review Board (STUDY2015_00000113). The patients/participants provided their written informed consent to participate in this study. The animal study was reviewed and approved by for NIRS measurements on the gray seal, wild-caught juvenile animals were temporarily held in a managed facility, before release back to the wild. Procedures for capture, handling and housing of animals conformed to the Animals (Scientific Procedures) Act 1986, under the Sea Mammal Research Units’ Home Office license (no. 70/7806) and were performed by personnel deemed competent under EU directive. The dolphin experiment was performed at the at the Fundación Oceanogràfic Valencia, Spain, and approved by the Oceanogràfic Animal Care & Welfare Committee at the Fundación Oceanogràfic Valencia, Spain (OCE-10-20), and the Bureau of Medicine (BUMED, NRD1170).

## Author Contributions

AR, JM, AF, BS-C, and JK conceived the study, contributed to the writing of the manuscript, and provided comments and advice. AR, JM, and AF performed the experiments. AR, JM, and JK provided model code and results. AR and JM wrote the manuscript. All authors contributed to the article and approved the submitted version.

## Conflict of Interest

AF was employed by company Global Diving Research Sociedad Limitada. The remaining authors declare that the research was conducted in the absence of any commercial or financial relationships that could be construed as a potential conflict of interest.

## Publisher’s Note

All claims expressed in this article are solely those of the authors and do not necessarily represent those of their affiliated organizations, or those of the publisher, the editors and the reviewers. Any product that may be evaluated in this article, or claim that may be made by its manufacturer, is not guaranteed or endorsed by the publisher.
